# Suprarenal interrupted inferior cava with azygos continuation in a patient with pulmonary embolism: a case report

**DOI:** 10.1093/ehjcr/ytae583

**Published:** 2024-10-29

**Authors:** Ehsan Shahverdi, Ramtin Keshavarzi, Mohammad Mazandarani, Carsten Schneider, Mathias Lange

**Affiliations:** Department of Cardiology, Rhythmology, Angiology and Intensive Care Medicine, Heart Center Osnabrueck, Hospital Osnabrueck, Westphalian Wilhelms University of Muenster, Osnabrueck, Germany; Department of Cardiology, Rhythmology, Angiology and Intensive Care Medicine, Heart Center Osnabrueck, Hospital Osnabrueck, Westphalian Wilhelms University of Muenster, Osnabrueck, Germany; Department of Radiology, Hospital Osnabrueck, Westphalian Wilhelms University of Muenster, Osnabrueck, Germany; Department of Cardiology, Rhythmology, Angiology and Intensive Care Medicine, Heart Center Osnabrueck, Hospital Osnabrueck, Westphalian Wilhelms University of Muenster, Osnabrueck, Germany; Department of Cardiology, Rhythmology, Angiology and Intensive Care Medicine, Heart Center Osnabrueck, Hospital Osnabrueck, Westphalian Wilhelms University of Muenster, Osnabrueck, Germany

**Keywords:** Interrupted inferior vena cava, IVC, Azygos, Pulmonary embolism, Case report

## Abstract

**Background:**

Interrupted inferior vena cava (IVC) is a rare developmental defect characterized by azygos continuation following failure of fusion of one or more of the component parts of the embryological IVC. It occurs in approximately one in 5000 of the general population. It is usually an isolated finding and is generally asymptomatic.

**Case summary:**

A 42-year-old patient presented for further clarification of syncope with a 4-day history of persistent exertional dyspnoea. Computed tomography revealed a central pulmonary artery embolism on both sides, with prominent azygos and hemiazygos veins and paravertebral collaterals. For further clarification, a magnetic resonance imaging (MRI) scan was performed, where hypoplasia or stenosis of the inferior suprarenal vena cava was noted. There were collaterals of the azygos and hemiazygos veins. The finding was consistent with the picture of a venous abnormality in the sense of an ‘interrupted IVC (suprarenal) with azygos continuation’.

**Discussion:**

In the case of an isolated interrupted IVC, the patient is usually asymptomatic, and the vascular anomaly itself does not mandate any treatment. This variation should be considered, especially in young patients with deep venous thrombosis (DVT) when no other reason for thromboembolism is evident. To prevent vascular events such as DVT or pulmonary embolism, lifelong anticoagulation therapy is recommended. An interrupted IVC can also cause procedural difficulties during right heart catheterization, electrophysiological studies, cardiopulmonary bypass surgery, femoral vein catheter advancement, IVC filter placement, and temporary pacing through the transfemoral route.

**Learning points**

- In instances of an isolated interrupted inferior vena cava (IVC), the patient typically shows no symptoms, and the vascular abnormality itself does not require any intervention.
- Nonetheless, this variation can lead to venous insufficiency in the lower extremities, increasing the risk of thromboembolic disorders. This possibility should be taken into account, particularly in younger patients with deep venous thrombosis (DVT) when no other cause for the thromboembolism is apparent.
- To avert vascular incidents like DVT or pulmonary embolism, long-term anticoagulation therapy is advised.
- An interrupted IVC may also lead to challenges during right heart catheterization, electrophysiological assessments, cardiopulmonary bypass surgery, femoral vein catheter advancement, IVC filter placement, and temporary pacing via the transfemoral approach.
- The diagnosis should be emphasized in hospital discharge summaries.

## Introduction

The absence of the hepatic segment of the inferior vena cava (IVC) with azygos continuation, also known as azygos continuation of the IVC, is a rare vascular anomaly with a prevalence of ∼0.2–3%.^1^ The most common pattern is azygos continuation with a normal cardiac position and normal position of abdominal viscera. This anomaly can be detected in the prenatal period^2^ or incidentally in the elderly.^3^ Isolated interrupted IVC with azygos continuation has been rarely reported.^4,5^ Here, we report the diagnosis of interrupted IVC with azygos continuation in a patient with central pulmonary artery embolism.

## Summary figure

**Table:** 

**Day 1**
A 42-year-old patient presented for further clarification of syncope, with a 4-day history of persistent exertional dyspnoea.
The laboratory results showed an increased D-dimer level.
CT revealed a central pulmonary artery embolism on both sides (saddle embolism) with very prominent azygos and hemiazygos veins, accompanied by strong paravertebral collaterals.
**Day 3**
On sonography, an unclear mass in the right kidney was noted, which was most likely cystic. For further clarification, magnetic resonance imaging was organized.
**Day 6**
In the MRI, there was no evidence of a tumorous or inflammatory process around the right kidney. Renal atrophy on the left side was noted. Additionally, hypoplasia or stenosis of the inferior suprarenal vena cava was observed. There were collaterals of the azygos and hemiazygos veins. The findings were consistent with the picture of a venous abnormality described as an ‘interrupted IVC (suprarenal) with azygos continuation’.
Cardiac MRI did not reveal any abnormalities or signs of cardiomyopathy.
**Day 7**
The day of discharge.

## Case presentation

A 42-year-old patient presented for further clarification of syncope with a 4-day history of persistent exertional dyspnoea. He reported a mild retrosternal burning sensation radiating caudally below the left and right costal arches. There was no history of trauma, diabetes, or surgery. The patient had no connective tissue disorders or other systemic anomalies and no significant family history of disease.

Physical examinations revealed tachypnoea with a respiratory rate of 28/min and a blood pressure of 170/110 mmHg. The rest of the physical examination was unremarkable. On the day of admission, the electrocardiogram showed sinus tachycardia with a heart rate of 110 b.p.m.

### Laboratory tests showed an increased D-dimer without evidence of thrombophilia (*Table 1*)

**Figure 1:**
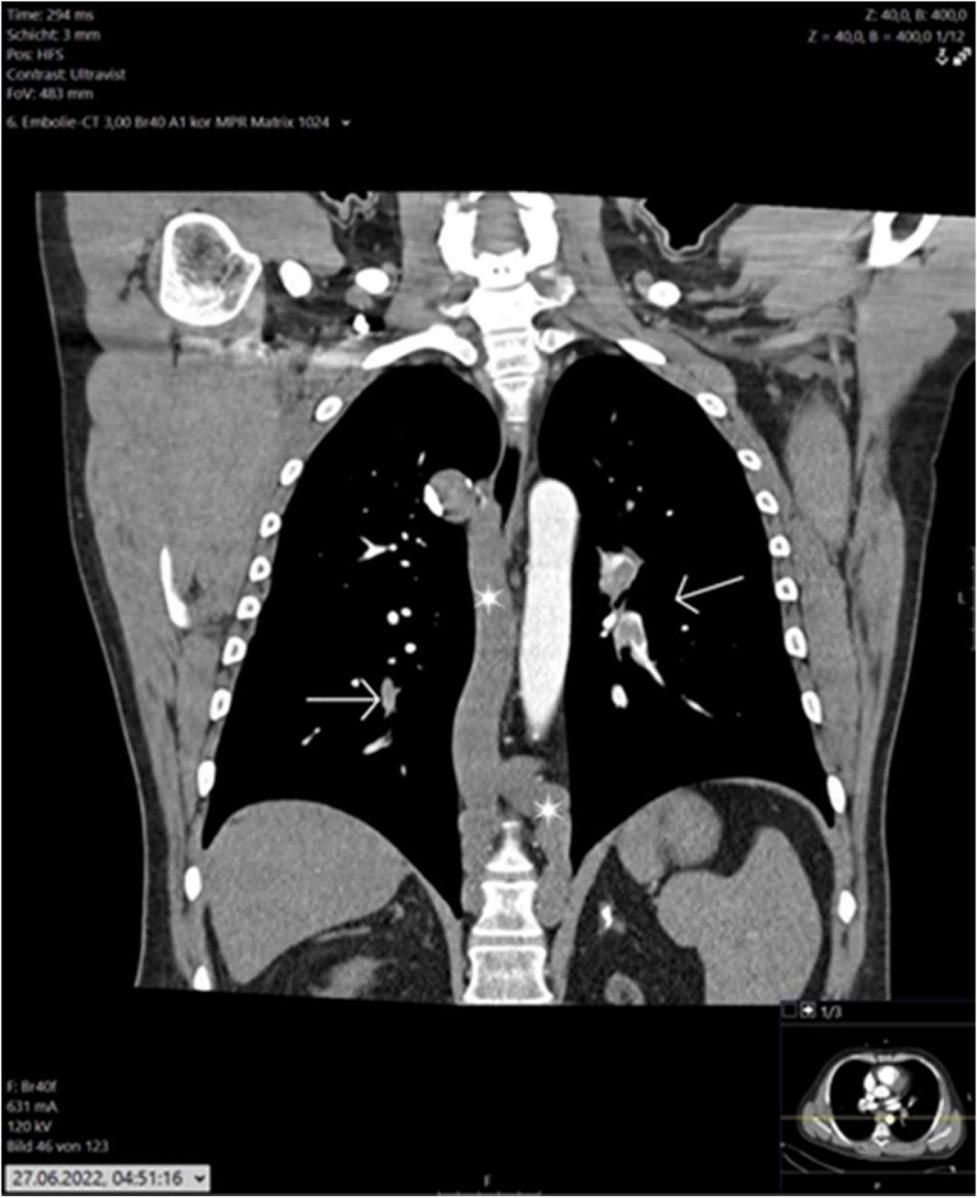
Dilated azygos and hemiazygos veins (asterisk), pulmonary embolism is also shown here (arrows).

Computed tomography (CT) of the head, as part of syncope clarification, showed no abnormalities. In the case of elevated D-dimers and sinus tachycardia to rule out pulmonary arterial embolism, a CT pulmonary angiography/angiogram was performed. It revealed a central pulmonary artery embolism on both sides (saddle embolism) without infarct, pneumonia, or CT morphological signs of cor pulmonale. Computed tomography revealed a very prominent azygos and hemiazygos vein with strong paravertebral collaterals (*Figure 1*). Compression sonography of the leg veins showed compressible veins and good flow on Doppler sonography, with no evidence of deep venous thrombosis (DVT). Echocardiography initially showed right heart strain, with normal left ventricular pump function, no regional wall motion abnormalities, and no significant valvular insufficiency.

**Table 1:** Laboratory results of the patient

	Unit	Reference range	
WBC	Tsd/µL	4.3–10	10.02 (+)
RBC	Mio/µL	4.50–5.90	5.26
Haemoglobin	g/dL	14.0–17.5	16.4
Haematocrit	%	40–52	47
MCV	fl	82–101	90
MCH	pg	27–34	31
MCHC	g/dL	31.5–36.0	35
Platelets	Tsd/µL	140–440	134 (−)
APC1-FV	s	128–173	178.9
MTHFR mutation PCR		Normal	Heteroz.
Factor V-Leiden mutation		Normal	Normal
Prothrombin		Normal	Normal
Procalcitonin	ng/mL	<0.5	0.44
Homocysteine	µmol/L	4.9–15.0	23.00 (+)
INR			1.0
AT III	%	>70	92
D-Dimer	mg/L	<0.5	2.73 (+)
aPTT	s	25.1–37.7	44 (+)
Sodium	mmol/L	136–145	142
Potassium	mmol/L	3.5–4.5	4.4
Magnesium	mmol/L	0.74–0.99	0.68
AST	U/L	<35	39 (+)
ALT	U/L	<50	61
GGT	U/L	<55	40
Creatinine	mg/dL	0.70–1.30	1.33
GFR	mL/min	>90	65
C-reactive protein	mg/dL	<0.3	1.1
von Willebrand factor	%	56–162	158.9
Lupus anticoagulant	%	50–187	168
Factor VIII	Ratio		1.06 (N)
Factor II	sec	30.4–45.3	54.2 (+)
Factor V	sec	27.7–33.5	37.7 (+)
Factor IX	Ratio	< 1.2	1.44 (+)
Factor XII	sec	30.4–45.3	44.4
Protein C	%	60–168	145.7
Protein S antigen	%	70–120	111
APC2-FV	%	70–140	74
APC-FV ratio	%	70–120	135.5 (+)
aPTT - lupus sensitive	%	70–150	82
aPTT FSL - lupus mix	%	70–140	>150 (+)
Platelet morphology	%	64.7–115.3	118.5 (+)
Fibrinogen	s	68–91	69.7
FT3	Ratio	0.86–1.10	0.90
FT4	sec	25–31	32.0 (+)
TSH 3 Ultra	sec	25–31	28.0
Lipoprotein (a)			Thrombocyte anisocytosis (*N*)
Cardiolipin IgG	mg/dL	180–350	363 (+)
Cardiolipin IgM	pg/mL	2.3–4.2	3.91
Anti-beta-2 Glycoprotein	ng/dL	0.89–1.76	1.25
ß2 Glycoprotein IgM	µIE/mL	0.55–4.78	3.04
NT-proBNP	g/L	<0.3	<0.10
Troponin high-sensitivity	GPL-U/mL	<10	1.2

ALT, alanine transaminase; APC-FV, activated protein C resistance - Factor V; AST, aspartate transaminase; GFR, glomerular filtration rate; GGT, gamma-glutamyl transferase; INR, international normalized ratio; MCH, mean corpuscular haemoglobin content; MCHC,mean corpuscular haemoglobin concentration; MCV, mean corpuscular volume; NT-proBNP: N-terminal Prohormone of Brain Natriuretic Peptide; PTT, partial thromboplastin time; RBC, red blood cell; TSH, thyroid-stimulating hormone; WBC, white blood cell.

Sonographically, an unclear mass in the right kidney was noted, which was most likely cystic. For further clarification, magnetic resonance imaging (MRI) was organized.

**Figure 2:**
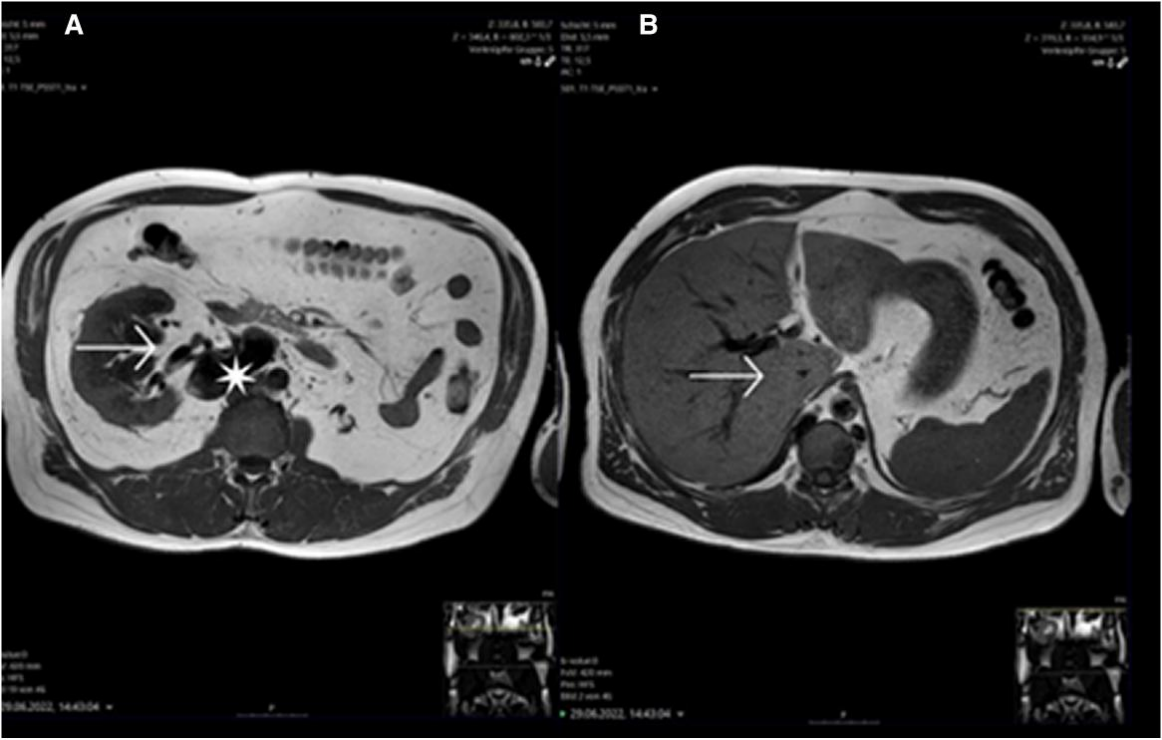
(*A*) Axial T1 shows the right renal vein (arrow) draining into a tortuous and dilated paravertebral collateral (asterisk). (*B*) Hypoplastic inferior vena cava at suprarenal level.

The MRI showed a well-defined lobulated mass medial to the right kidney with slow venous flow on axial T2 imaging, consistent with distended collateral veins immediately inferior to complete stenosis of the IVC, with caudal segments of the IVC preserved. The distended collateral veins communicated posteriorly with the ascending lumbar veins. The findings were consistent with the picture of a venous abnormality in the sense of an ‘interrupted IVC (suprarenal) with azygos continuation’ (*Figure 2*). Here, renal atrophy on the left side was detected. In addition, hypoplasia or stenosis of the inferior suprarenal vena cava was noted. There were collaterals of the azygos and hemiazygos veins.

### Cardiac magnetic resonance imaging did not reveal any abnormalities or cardiomyopathy

On the day of discharge, the patient presented with no thoracic discomfort or dyspnoea symptoms. An echocardiogram performed showed normal right ventricular function with no signs of right heart strain.

Considering a persistent high risk of recurrence of DVT and pulmonary embolism, the patient was discharged with a recommendation to continue anticoagulation therapy with apixaban 5 mg two times a day for life.

## Discussion

Interrupted IVC results from the failure of fusion of the component parts of the embryological IVC and may occur at any level. The IVC is composed of four segments: hepatic, pre-renal, renal, and post-renal. These segments arise from the formation, fusion, and regression of paired cardinal veins. The hepatic segment is formed from the hepatic veins, the pre-renal segment from the sub-cardinal vein, and the post-renal segment from the supra-cardinal vein, while the renal segment is formed from the sub-cardinal and supra-cardinal anastomosis. The azygos system is formed from the supra-cardinal veins. The hepatic segment is subdivided into cranial and caudal extensions. The caudal extension fuses with the pre-renal segment to provide caudal continuity, while the cranial extension receives the hepatic veins and drains into the right atrium. Failure of the pre-renal segment and the caudal extension of the hepatic segment to fuse results in interrupted IVC at this level, and venous flow beyond this point continues via a dilated azygos system, which drains into the right atrium via the superior vena cava (SVC). The cranial extension of the hepatic segment drains directly into the right atrium.^6^ Interruption of the IVC with azygos continuation is rare. Its prevalence is <0.3% among normal patients without congenital heart disease.^7^ In 90% of cases, it occurs as an isolated anomaly, although it may be associated with cardiac or splenic abnormalities.^2^ In the present study, we reported a case with no other anomalies, which is also noted in some other studies, such as the Trubac report, which documented a case with congenital interruption of the IVC with hemiazygos continuation but without any other anomalies.^8^ However, Colak *et al*.^9^ reported a patient with interrupted IVC and an atrial septal defect. Kim *et al*.^10^ reported interrupted IVC with hemiazygos continuation in an adult with a left single coronary artery. When occurring in isolation, it is usually asymptomatic with no clinical signs, and the vascular anomaly itself does not mandate any treatment. This variation can cause venous insufficiency of the lower limbs with potential thromboembolic disease, which can lead to pulmonary embolism.^11^ This should be considered especially in young patients with DVT when no other reason for thromboembolism is evident. Although, in the current study, DVT was not reported, pulmonary embolism was the first manifestation. After ruling out all risk factors for DVT, we assume that the abnormality is the primary cause of pulmonary embolism. To prevent recurrence of DVT or pulmonary embolism, lifelong anticoagulation therapy was recommended.

Anticoagulation is the mainstay of therapy for patients with acute pulmonary embolism, while systemic thrombolysis is reserved for those with haemodynamic instability. Treatment with systemic thrombolysis is associated with an increased risk of major bleeding (13%) and stroke (3%).^12^ In high-risk pulmonary embolism (PE) patients, the beneficial effects of thrombolysis outweigh the risk of bleeding. In intermediate-risk patients, however, the beneficial effects of thrombolysis do not balance the bleeding risk. While one-third of PE patients have contraindications to thrombolysis, even in patients eligible for thrombolysis, up to two-thirds do not receive the treatment.^13^ Surgical embolectomy may be an option for a fraction of these patients; however, the poor preoperative state together with a high degree of comorbidity makes the majority poor candidates for surgery. This leaves a substantial proportion of PE patients undertreated. Novel catheter-based approaches may provide effective thrombus removal without the risks associated with systemic thrombolytic or surgical treatment.^12^ Numerous new techniques have been developed and refined during the last decade. An interrupted IVC can also cause procedural difficulties during right heart catheterization, making this method inadvisable in patients with a VC anomaly. Deep venous thrombosis and pulmonary embolism are not the only reported symptoms; other symptoms, such as cardiac arrhythmia, have also been reported.^14^

The dilated azygos/hemiazygos system shown by chest or abdominal X-ray films can be misinterpreted as a mediastinal or retroperitoneal neoplasm, lymphadenopathy, or aortic dissection.^14^ The chest radiography in our recent case was normal with no mass.

Venostasis due to pathological conditions such as acquired obstruction of the IVC or SVC, right heart failure, portal hypertension, or pregnancy can present similarly to azygos/hemiazygos continuation of the IVC. In the recent case, the N-terminal prohormone of brain natriuretic peptide was elevated, either in the context of pulmonary embolism or venostasis.

An interrupted IVC can cause procedural difficulties during right heart catheterization, electrophysiological studies, cardiopulmonary bypass surgery, femoral vein catheter advancement, IVC filter placement, and temporary pacing through the transfemoral route.^7^ Hardwick *et al*.^15^ reported interrupted IVC as a high-risk anatomy for right thoracotomy.

## Conclusion

An isolated interrupted IVC is usually asymptomatic, and the vascular anomaly itself does not mandate any treatment. It may indicate other associated variations or anomalies, especially heart defects, which should be considered. This variation can cause venous insufficiency of the lower limbs with potential thromboembolic disease. This should be particularly noted in young patients with DVT when no other reason for thromboembolism is evident. To prevent recurrence of DVT or pulmonary embolism, lifelong anticoagulation therapy is recommended. The dilated azygos/hemiazygos system shown by chest or abdominal X-ray films can be misinterpreted as a mediastinal or retroperitoneal neoplasm, lymphadenopathy, or aortic dissection. An interrupted IVC can also cause procedural difficulties during right heart catheterization, electrophysiological studies, cardiopulmonary bypass surgery, femoral vein catheter advancement, IVC filter placement, and temporary pacing through the transfemoral route. Therefore, the diagnosis should be highlighted in hospital discharge summaries. Venostasis due to pathological conditions such as acquired obstruction of the IVC or SVC, right heart failure, portal hypertension, or pregnancy can present similarly to azygos/hemiazygos continuation of the IVC.

## Data Availability

The data underlying this article cannot be shared publicly due to privacy of individuals who participated in the study. The data will be shared on reasonable request to the corresponding author.

## Lead author biography

Mathias Lange currently works at the Clinic of Cardiology, Klinikum Osnabrück. He is the head of the Structural Heart Department. Mathias does research in Cardiology: Their current project is ‘Left Atrial Appendage Closure - Clinical Follow Up’ and other topics such as transcatheter mitral- and tricuspid valve repair.

**Consent:** The authors confirm that consent for submission and publication of this case report has been obtained from the patient in line with the COPE guidance.

**Funding:** None declared.
